# Supplementary material for: Reference genes for mesangial cell and podocyte qPCR gene expression studies under high-glucose and renin-angiotensin-system blocker conditions
Source: PLoS One. 2021 Jul 9;16(7):e0246227. doi: 10.1371/journal.pone.0246227 (PMC8270477; doi:10.1371/journal.pone.0246227)
Supplement: Attachment — Submitted filename: Response to Reviewers.docx [file pone.0246227.s001.docx]

Dear Editor, Dr. Muhammad Shareef Masoud

We would like to thank you for the opportunity to revise the manuscript entitled "*Reference genes for mesangial cell and podocyte qPCR gene expression studies under high-glucose and renin-angiotensin-system blocker conditions*". We have carefully considered the comments and concerns of the Reviewers and have made changes to the manuscript according to the suggestions, which are considered point by point below (italicized) and highlighted in red in the manuscript. The Reviewers' comments were very constructive and helped to improve the manuscript.

The revised version of the manuscript has been read and approved by all authors, and each author has provided the attention necessary to ensure the integrity of the work. We kindly ask you to reconsider the manuscript in the current form and hope that you judge it suitable for publication in *PLOS ONE*.

Kind Regards,

Dr. Mirian Aparecida Boim

**Reviewer #1**

- A certain view is implied by the authors regarding to this study but they could be more explicit. The authors take a rather narrow view of data publication, which I think hinders their analyses.
  - *We would like to thank this Reviewer for the time dedicated to evaluate this manuscript. We have reviewed the introduction and discussion sections to strengthen the background and rationale of our study, especially including previous data published from other groups.*
- Results should be reconfirmed with other assay as well.
  - *We appreciate the Reviewer’s comment. Literature has shown that probe-based PCR can be adequately reproduced by SYBR Green real-time PCR, delivering highly comparable results with TaqMan and other quantitative gene expression methods (Tajadini 2014,* [*10.4103/2277-9175.127998*](https://dx.doi.org/10.4103%2F2277-9175.127998)*; Arikawa 2008, 10.1186/1471-2164-9-328). Other than that, our melting curve analysis showed neither unspecific products nor primer dimers. We also made sure to use proper negative controls for all reactions performed. Multiple studies specifically on reference genes have been performed based on SYBR Green assays only (Zhang 2017, 10.3892/ol.2017.7002; Adeola 2018, 10.4314/ejhs.v28i6.9; Hashemi 2021, 10.1016/j.bbrc.2012.09.009; Nygard 2007, 10.1186/1471-2199-8-67; Ahn 2008, 10.1186/1471-2199-9-78; Bokhale 2020, 10.1016/j.dib.2020.105750). Considering all this information, we believe our results are reliable.*
- Manuscript writeup should improve.
  - *We appreciate the Reviewer's suggestion. We submitted the manuscript to standard English grammatical revision before resubmission of this revised manuscript to PLOS ONE.*
- Figure legends could not show proper description, So its better to write in detail.
  - *We agree with the Reviewer’s comment, and we have altered the figure legends by including details, as suggested.*

**Reviewer #2**

- In this manuscript entitled Reference genes for mesangial cell and podocyte qPCR gene expression studies under high-glucose and renin-angiotensin-system blocker conditions analyze six genes (HPRT, ACTB, PGAM-1, GAPDH, PPIA, and B2M) using five software applications and the ΔCt method to determine that the best genes to be used for mesangial cell studies with high glucose and angiotensin receptor II blocker are HPRT and ACTB, while in the same conditions, the best combination of genes for podocyte gene expression normalization is HPRT. The study described in the manuscript is interesting however the manuscript can be considered for publication only if the following issues are addressed.
  - *We would like to thank this Reviewer's careful reading and attention to our work. We have addressed the issues as the following:*
- Why authors select these six genes (HPRT, ACTB, PGAM-1, GAPDH, PPIA, and B2M). Please elaborate in this article with references.
  - *We agree with the Reviewer’s comment. We have included the rationale of each gene included in our study in the Discussion section, with references.*
- Authors should also compare the results of syber green with probe based real time PCR
  - *We appreciate the Reviewer’s comment. Literature has shown that probe-based PCR can be adequately reproduced by SYBR Green real-time PCR, delivering highly comparable results with TaqMan and other quantitative gene expression methods (Tajadini 2014,* [*10.4103/2277-9175.127998*](https://dx.doi.org/10.4103%2F2277-9175.127998)*; Arikawa 2008, 10.1186/1471-2164-9-328). Other than that, our melting curve analysis showed neither unspecific products nor primer dimers. We also made sure to use proper negative controls for all reactions performed. Multiple studies specifically on reference genes have been performed based on SYBR Green assays only (Zhang 2017, 10.3892/ol.2017.7002; Adeola 2018, 10.4314/ejhs.v28i6.9; Hashemi 2021, 10.1016/j.bbrc.2012.09.009; Nygard 2007, 10.1186/1471-2199-8-67; Ahn 2008, 10.1186/1471-2199-9-78; Bokhale 2020, 10.1016/j.dib.2020.105750). Considering all this information, we believe our results are reliable.*
- Grammatical errors in all parts of manuscript.
  - *We appreciate the Reviewer's suggestion. We submitted the manuscript to standard English grammatical revision before resubmission of this revised manuscript to PLOS ONE.*

*We are grateful for the Reviewers' careful reading and attention to our work. Their suggestions were very relevant and contributed to improve our manuscript. We hope with these corrections and enhancements this manuscript can be considered eligible for publication in PLOS ONE.*
